# Tinea incognito skin lesions worsen after antifungal treatment: Atypical tinea appearing twice in a case: A case report

**DOI:** 10.1097/MD.0000000000043875

**Published:** 2025-08-15

**Authors:** Nenghan Zhang, Ruifeng Zhang

**Affiliations:** a Xi’an Jiaotong University Health Science Center, Xi’an, China; b Department of Dermatology, Hanzhong Central Hospital, Hanzhong, China.

**Keywords:** accompanied bacterial infection, atypical tinea, empirical treatment, folliculitis, fungal microscopy examinations, recurrent, tinea incognito

## Abstract

**Rationale:**

Atypical tinea lesions can exhibit diverse manifestations. Positive fungal microscopy results confirmed the diagnosis of tinea incognito and other forms of atypical tinea. Patients may concurrently suffer from fungal and bacterial coinfections, requiring physicians to carefully observe, accurately assess, and implement treatments that target both fungi and bacteria.

**Patient concerns:**

After a 10-month period of misdiagnosis, fungal elements were identified upon examination, suggesting a possible breakthrough in diagnosis. However, the worsening of skin lesions following antifungal treatment raises concerns regarding alternative diagnoses. Subsequent antifungal and antibacterial therapies have led to the resolution of facial skin lesions. However, the fungal and bacterial infections recurred after reusing the same skincare products. This relapse highlighted the potential role of external contaminants in disease recurrence. Fortunately, the infection was successfully eradicated with appropriate treatment.

**Diagnoses:**

Based on the positive fungal microscopy result, the patient was diagnosed with tinea incognito. However, the skin lesions worsened after the antifungal treatment. Given the emergence of multiple small papulopustular lesions, an additional diagnosis of folliculitis was established. Two weeks after achieving a clinical cure with treatment, the skin lesions recurred. The physician determined that this was caused by a facial infection from contaminated skincare products, leading to a rediagnosis of atypical tinea and folliculitis.

**Interventions:**

The initial treatment involved antifungal therapy alone. Antibacterial therapy was administered after the skin lesions worsened. When the lesions recurred 2 weeks following their resolution and antifungal monotherapy failed to achieve complete clearance, antibacterial treatment was reintroduced.

**Outcomes:**

After the initial combined antifungal and antibacterial therapy, the skin lesions resolved completely. Two weeks later, when the lesions recurred, antifungal treatment was administered first, followed by antibacterial therapy. This sequential approach ultimately cleared the lesion.

**Lessons:**

Diagnosis of tinea incognito relies on positive fungal microscopy results. When papulopustular lesions emerge following antifungal treatment, the possibility of concurrent bacterial infections should be considered. Essential therapeutic interventions require both antifungal and antibacterial therapies. Contaminated skincare products must be identified and eliminated to prevent the recurrence of fungal and bacterial infections. Empirical therapy is warranted when prompt therapeutic decisions are needed pending delayed fungal/bacterial test results.

## 1. Introduction

Fungal skin infections are commonly acquired by contact with fungus-infected animals or contaminated materials. Typical tinea corporis lesions present as well-defined ring-shaped erythema with papules, vesicles, and scales. Tinea incognito (TI), a form of atypical tinea, refers to lesions that appear atypical owing to the topical application of corticosteroids or immunosuppressants.^[1]^ The diagnosis of TI requires repeated fungal microscopy examinations, especially when fungal culture is unavailable.^[2]^ Once TI is confirmed, antifungal therapy should be immediately initiated.^[3]^ How should the management approach be adjusted if the skin lesions worsen during antifungal therapy? Fungal skin infections may also be associated with bacterial infection. Close attention should be paid to the progression of skin lesions given the absence of bacterial culture. Definitive treatment should involve a combination of antifungal and antibacterial therapies. Even after successful treatment, patients may experience recurrence with atypical dermatophytosis.^[4]^ Therefore, the prevention of recurrence should be prioritized.

## 2. Case presentation

### 2.1. Case history

A 25-year-old female, presented with facial erythematous papulopuritic lesions that had been itching for 10 months. The results of previous fungal microscopy tests were negative. Consequently, she was diagnosed with dermatitis and treated with oral cetirizine tablets, tranilast capsules, desloratadine tablets, vitamin C, vitamin E, acrivastine capsules, topical tacrolimus, and topical corticosteroid cream. However, these treatments were ineffective.

Upon further inquiry into the patient’s medical history, it was revealed that the patient had come into contact with a pet dog undergoing hair epilation 10 months before symptom onset. The patient had no history of hypertension or diabetes, and was otherwise healthy.

**Figure 1.**
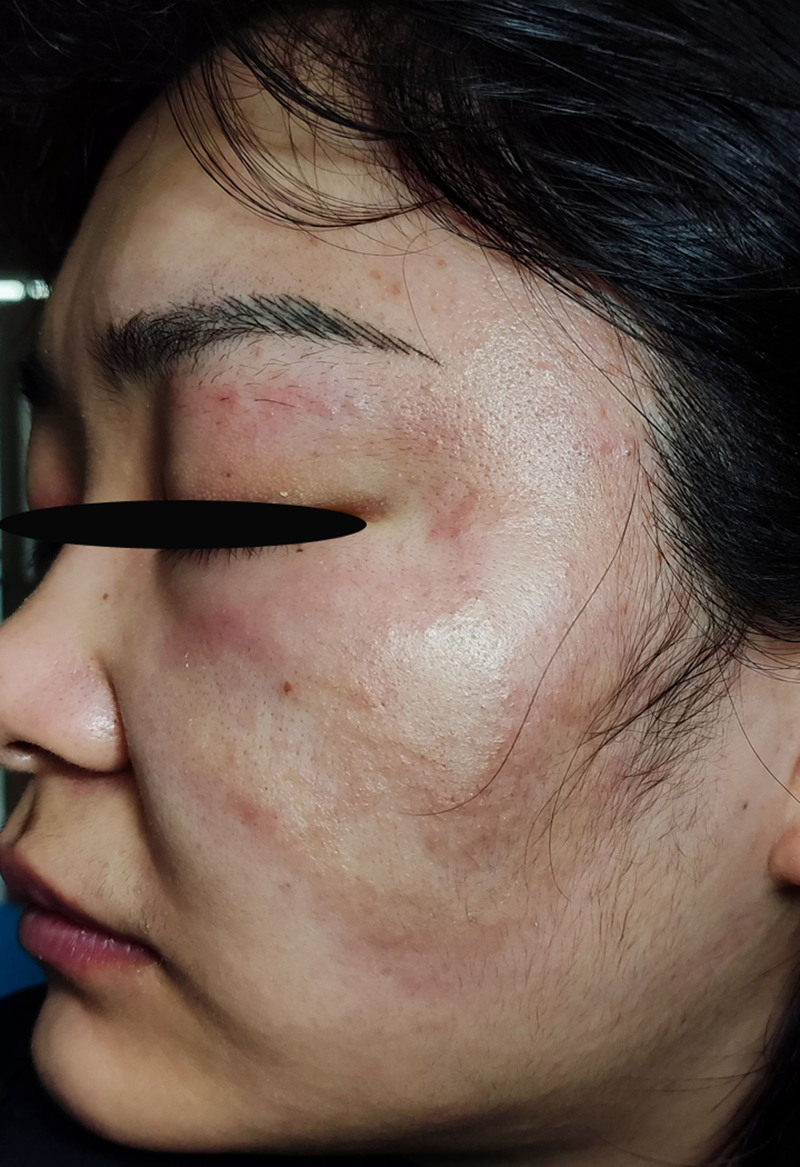
Dark erythema and papules. No obvious desquamation.

**Figure 2.**
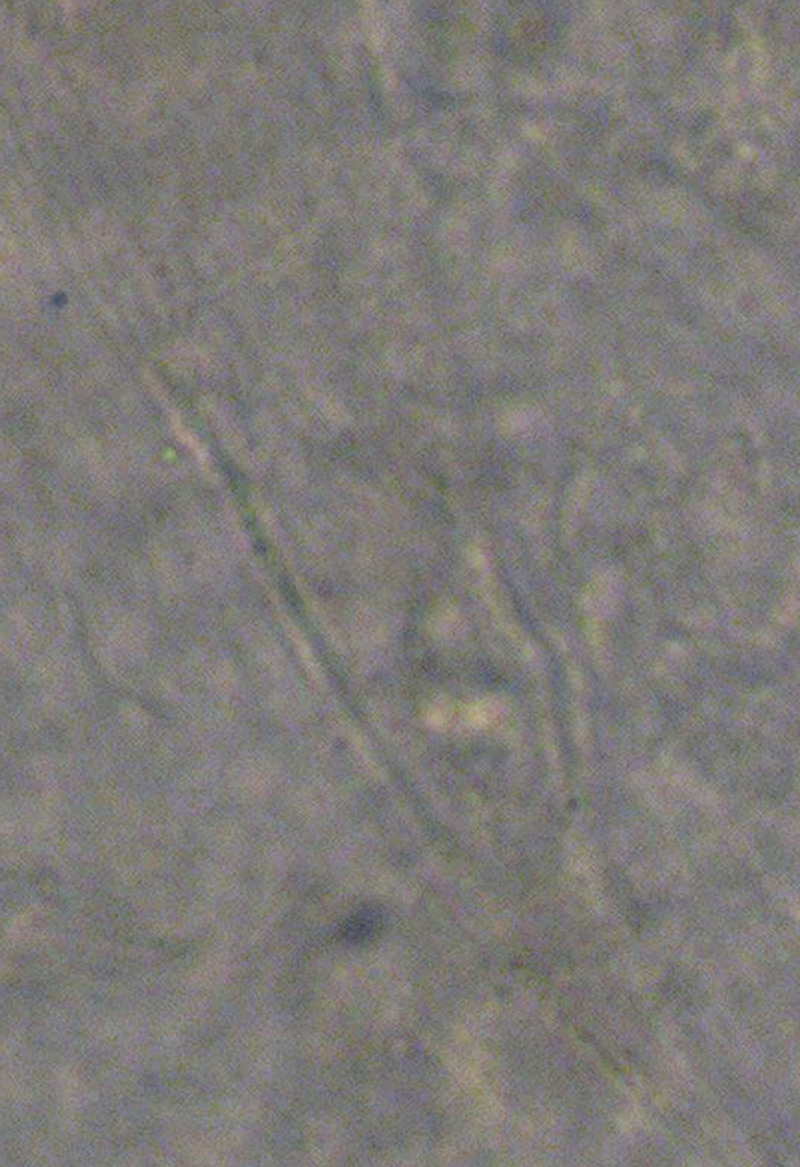
Atypical hyphae. Potassium hydroxide (KOH) 400×.

Physical examination revealed dusky erythematous patches and papules on the left cheek, with some lesions demonstrating sclerodermoid texture accompanied by mild swelling on palpation. The edges of the erythema were slightly raised without obvious desquamation (Fig. 1). Repeat fungal microscopy yielded positive results (Fig. 2), which led to a diagnosis of facial TI.

### 2.2. Therapeutic intervention and outcomes

#### 2.2.1. Phase I: Therapeutic intervention and outcomes

Following comprehensive evaluation, including a complete blood count, rapid C-reactive protein test, hepatic/renal function assessments, and blood glucose/lipid profiling, all parameters remained within normal ranges. The patient was prescribed itraconazole capsules 100 mg orally once daily and acrivastine capsules 8 mg orally thrice daily. Naftifine–ketoconazole cream was applied twice daily.

**Figure 3.**
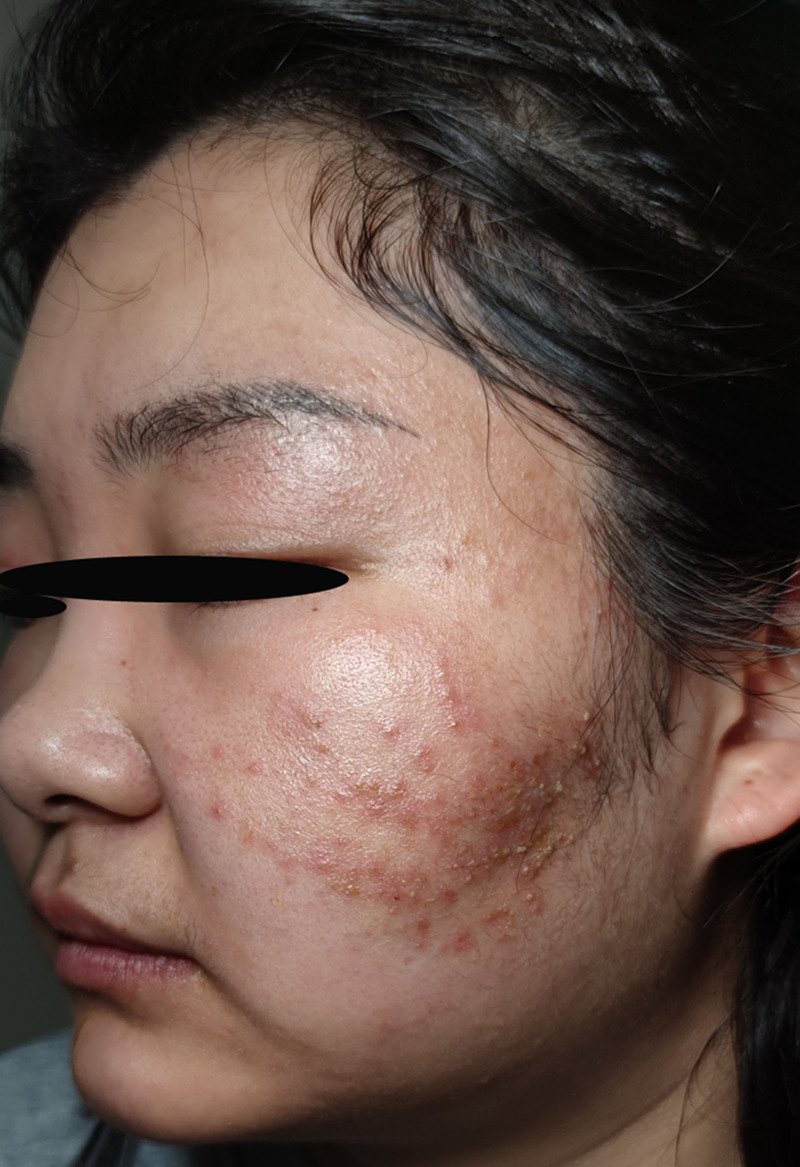
Multiple small pustules appeared.

After 1 week of therapy, baseline erythema showed improvement, and the preexisting mild induration and swelling had noticeably softened. However, multiple newly developed 2 to 3 mm papulopustular lesions emerged in the originally affected area (Fig. 3). An additional diagnosis was established on the basis of the findings of secondary bacterial folliculitis. The regimen was augmented with erythromycin enteric-coated capsules (250 mg) administered orally thrice daily. Fusidic acid cream was applied 3 times daily. This combined therapy was continued for 7 days.

**Figure 4.**
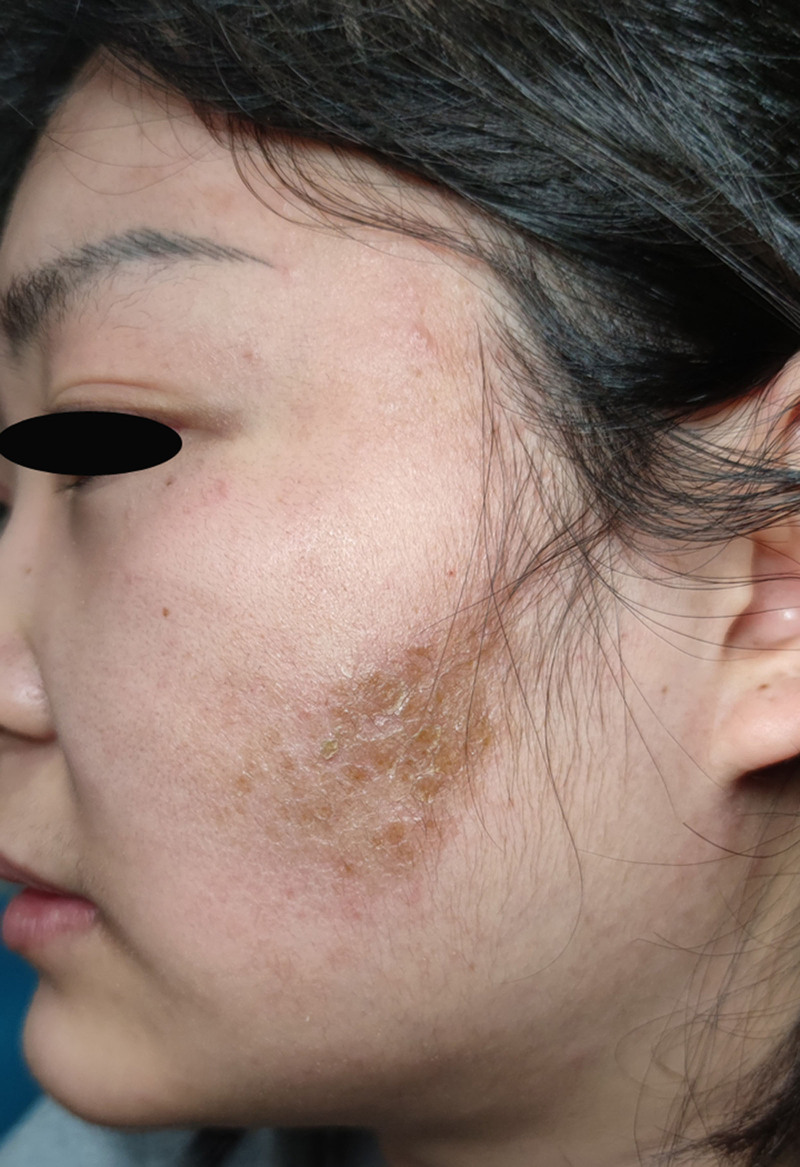
Yellow crusts.

Two weeks after the initial presentation, the erythematous pustular papules had resolved, with subsequent development of yellow crustation (Fig. 4). The established therapeutic regimen, comprising itraconazole, naftifine–ketoconazole cream, erythromycin enteric-coated capsules, and fusidic acid cream, was maintained for 7 additional days.

**Figure 5.**
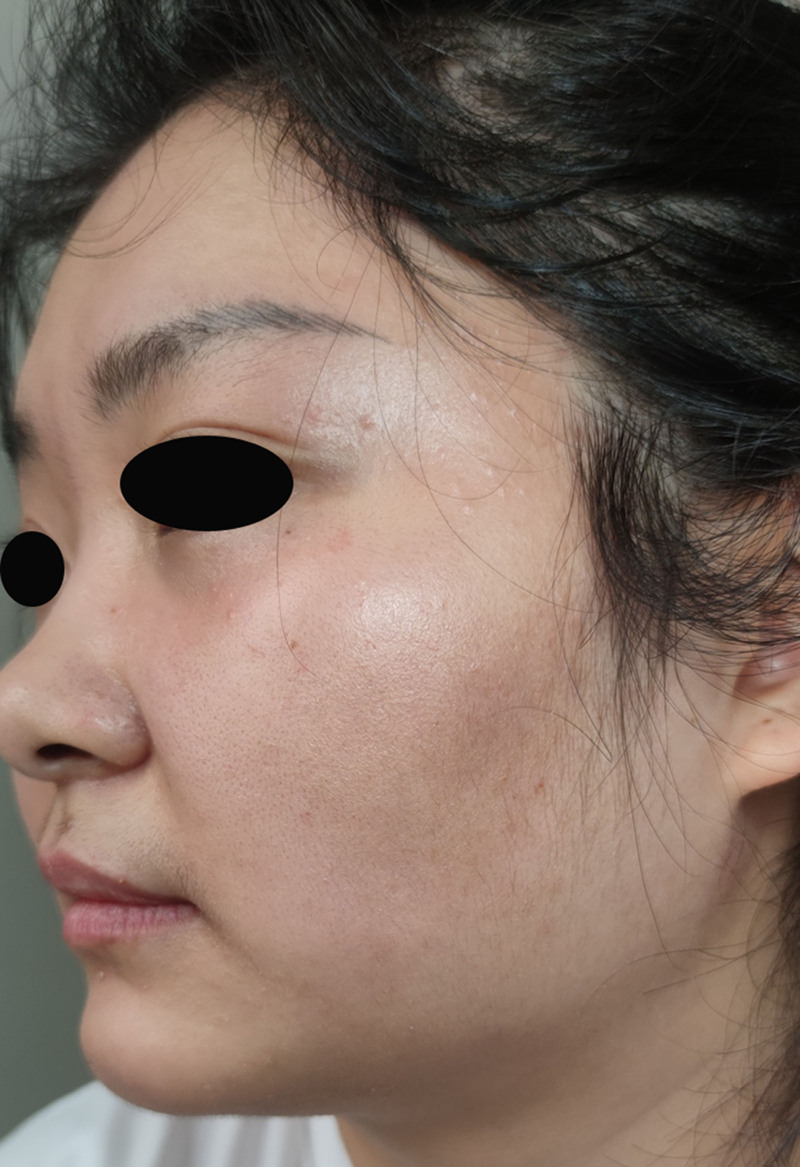
Slight local pigmentation and a few pimples.

Three weeks after the initial presentation, complete resolution of the original erythematous papules, pustules, and crusted lesions was observed (Fig. 5). The facial skin demonstrated restored integrity with minimal post-inflammatory hyperpigmentation and sparse residual papulopustular lesions. Therapeutic modifications included discontinuation of erythromycin and fusidic acid creams and continuation of itraconazole and naftifine–ketoconazole creams. This regimen was extended for an additional 7 days.

**Figure 6.**
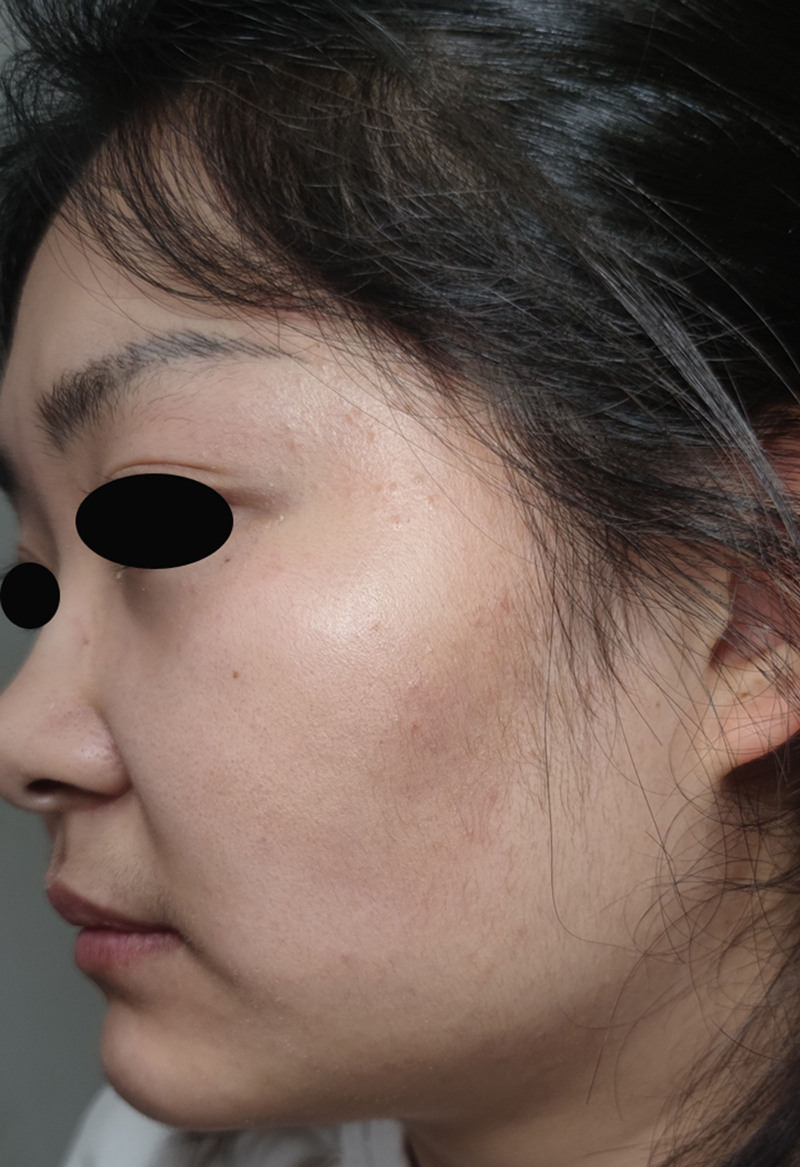
The skin was normally smooth and soft.

At the 4-week post-initial presentation, the primary cutaneous manifestations completely resolved. Dermatological examination revealed restored epidermal integrity, with physiological texture and elasticity (Fig. 6). Repeated fungal microscopy revealed the absence of hyphae or spores. All therapeutic interventions were discontinued, and clinical resolution was complete.

#### 2.2.2. Phase II: Therapeutic intervention and outcomes

**Figure 7.**
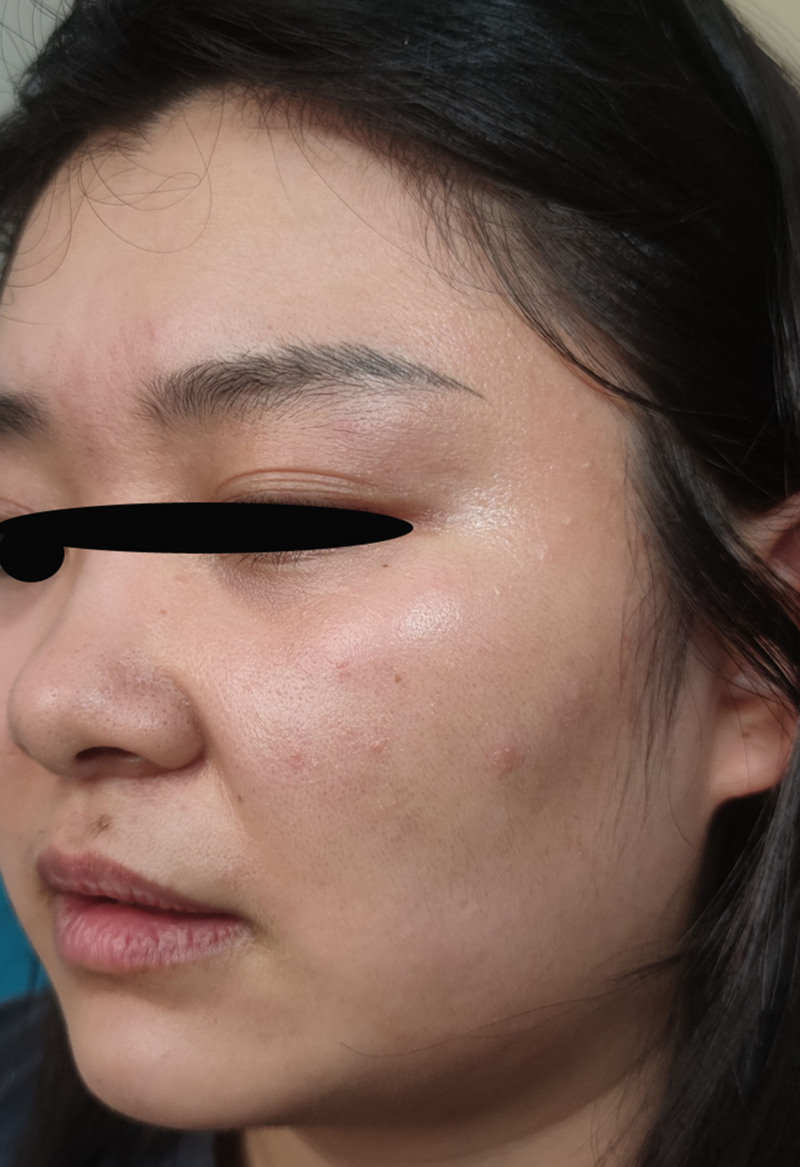
Redness, papules.

Six weeks after the initial visit, the patient revisited the hospital and presented with recurrent erythematous papulopruritic lesions involving the previously affected region (Fig. 7). Repeated potassium hydroxide preparation demonstrated the absence of fungal elements.

Upon investigating the cause of recurrence, the patient disclosed that she had repeatedly used skincare products within the preceding 14 days that had also been applied during the previous illness period. Considering the patient’s history of tinea incognito and the absence of topical corticosteroid or immunosuppressant use before this episode, the patient was diagnosed with atypical tinea. A 14-day antifungal protocol combining oral itraconazole (100 mg/day) with topical naftifine–ketoconazole cream (twice daily) was implemented.

**Figure 8.**
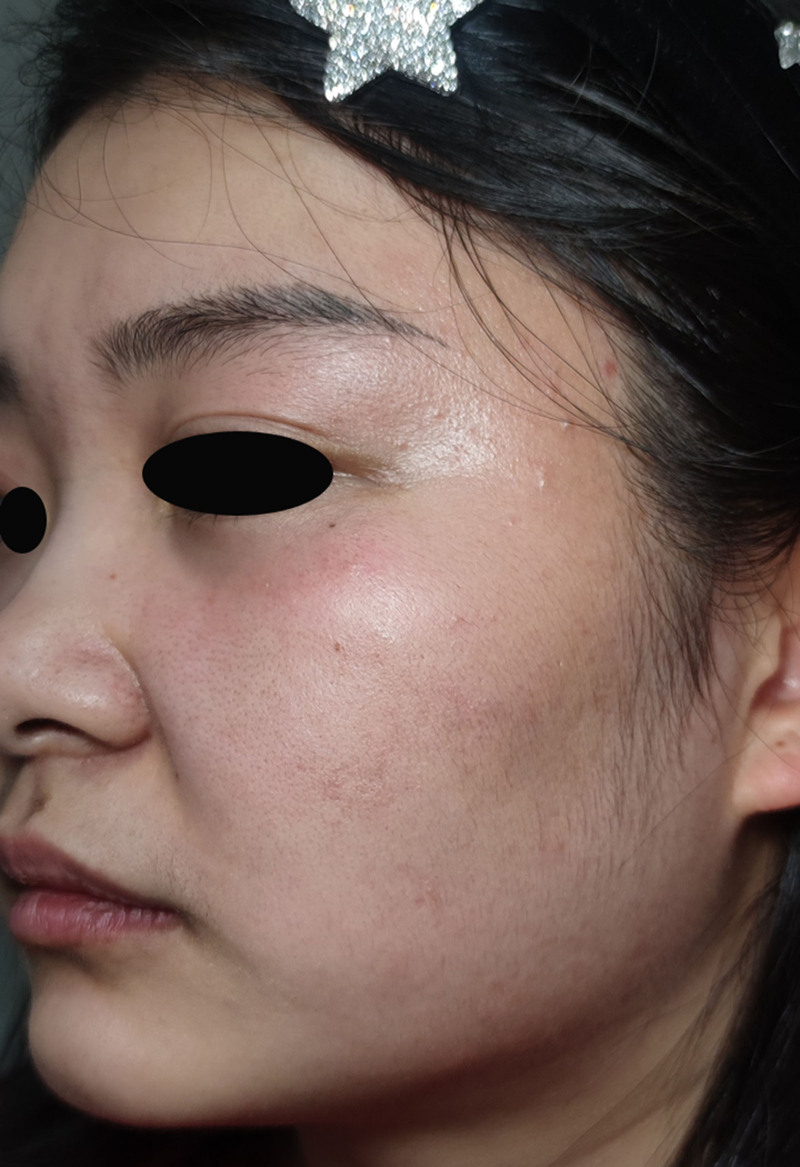
Erythema has become lighter, papules have decreased.

At the 8-week post-initial presentation (after 2 weeks of antifungal therapy), the patient exhibited significant clinical improvement, including resolution of the left facial erythema, reduced papules, and alleviated pruritus (Fig. 8). Antifungal therapy was subsequently extended to 14 days to prevent relapse.

**Figure 9.**
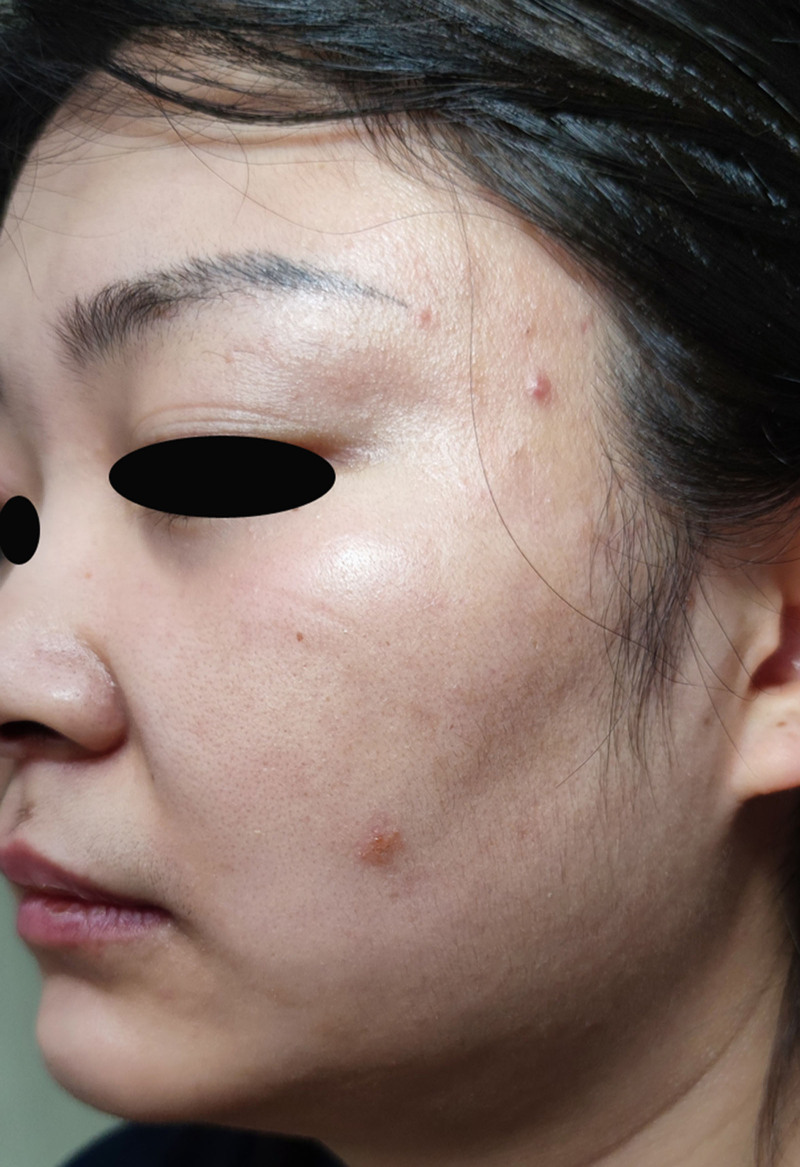
Small patches of erythema and follicular papules newly developed.

Ten weeks after the initial visit (following a completed second 4-week course of antifungal therapy), new small erythematous patches and follicular papules emerged on the patient’s face (Fig. 9). Results of fungal microscopy were negative. Considering the completion of 4 weeks of antifungal treatment, these newly emerged small erythematous papules were diagnosed as folliculitis. Antifungal medication was discontinued and a 2-week antibacterial regimen consisting of oral erythromycin enteric-coated capsules (250 mg twice daily) combined with topical fusidic acid cream (applied 3 times daily) was administered. At the 2-week follow-up, clinical examination confirmed complete resolution of facial erythema, papules, and pruritus.

**Figure 10.**
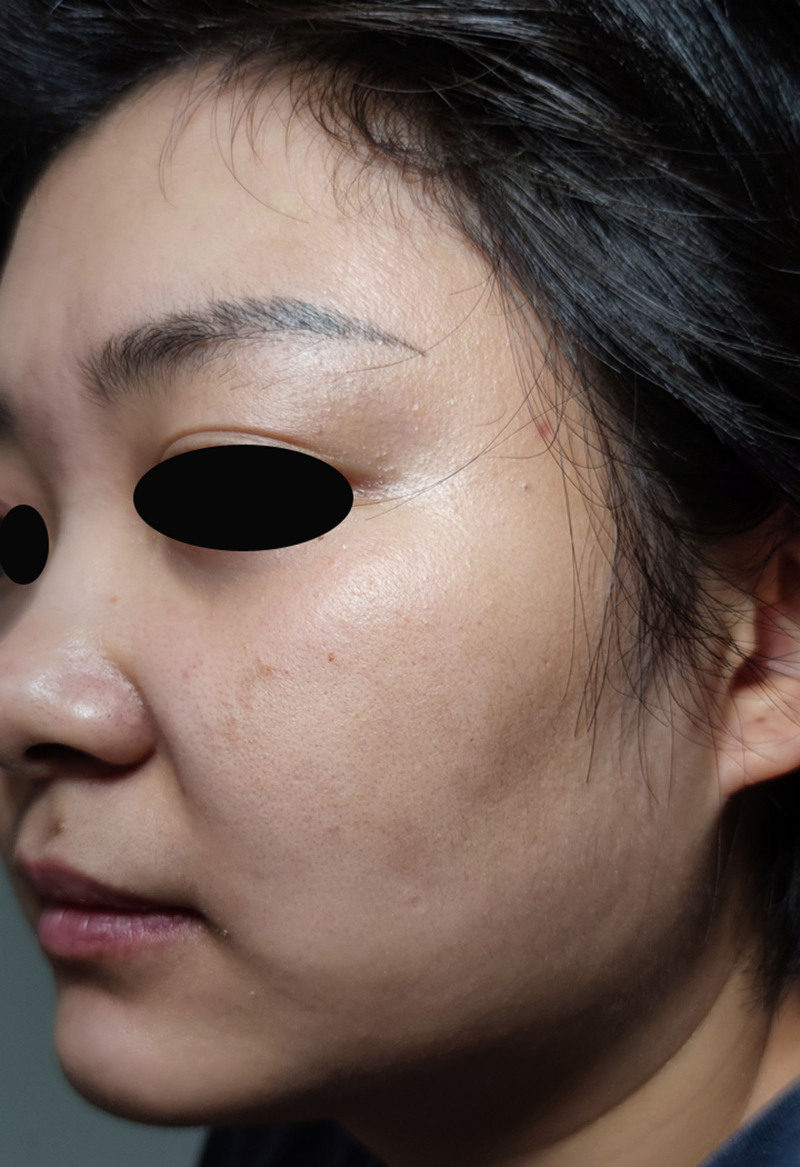
The skin was normally smooth and soft again.

At the 14-week follow-up after the initial visit, clinical examination confirmed complete resolution of the patient’s facial lesions, with restoration of normal skin integrity (Fig. 10). Subsequent telephone follow-ups over the next 3 months revealed no evidence of recurrence. The antimicrobial treatment regimens used are listed in Table 1.

**Table 1:** Antimicrobial therapeutic medications.

Week	1	2	3	4	5	6	7	8	9	10	11	12
Itraconazole	√	√	√	√			√	√	√	√		
Naftifine–ketoconazole cream	√	√	√	√			√	√	√	√		
Erythromycin enteric-coated capsules		√	√								√	√
Fusidic acid cream		√	√								√	√

## 3. Discussion

The patient’s clinical course and treatment response were characterized by the following key features.

### 3.1. Prolonged misdiagnosis and prior treatments

A 10-month medical history was marked by multiple misdiagnoses and ineffective therapies, including prior use of topical corticosteroid cream and tacrolimus ointment.

### 3.2. Zoonotic exposure and diagnostic challenges

Documented contact with a depilated pet dog. Initial fungal microscopy was negative; however, repeat testing revealed hyphae with atypical morphology, supporting fungal etiology.

### 3.3. Fungal–bacterial coinfection dynamics

- Pre-antifungal phase: Presenting with dark erythema and papules (no pustules).
- Post-antifungal response: Significant reduction in erythema and edema, confirming the antifungal efficacy.
- Secondary bacterial infection: Emergence of papulopustular lesions after antifungal therapy, likely due to disruption of cutaneous microbiota balance.
- Combination therapy: Resolution of pustules and skin lesions following concurrent antifungal and antibacterial regimens.

### 3.4. Recurrence linked to skincare products

Recurrence of lesions after reuse of skincare products from the prior illness period. Despite negative fungal microscopy results, clinical improvement after antifungal retreatment suggested a relapsed fungal infection.

### 3.5. Final recovery and follow-up

After additional antifungal therapy, the residual erythematous papules resolved completely, with subsequent antibacterial treatment. Three-month follow-up confirmed sustained remission without recurrence.

## 4. Conclusions

1. Accurate diagnosis of TI requires:

- A comprehensive medical history included contact with depilated animals and prior use of glucocorticoid/tacrolimus creams.
- Repeated fungal microscopy (critical for detecting hyphae/mycelia).

1. The erythematous lesions transformed into papulopustular eruptions after antifungal therapy: Does this indicate diagnostic or therapeutic errors or other underlying causes? Close observation revealed improvement in erythema and softening of the preexisting mild induration, confirming the efficacy and appropriateness of antifungal treatment. New-onset papulopustular lesions likely result from bacterial superinfection. Resolution following adjunctive antibacterial therapy validates this hypothesis, ultimately achieving complete clearance. This case underscores the critical importance of meticulous lesion assessment and timely empirical intervention, especially when timely therapeutic decisions are required and microbial examination results cannot be promptly obtained.
2. Management of lesion recurrence: Two weeks after the initial cure, erythematous papules with pruritus recurred on the patient’s face. Repeated potassium hydroxide preparation showed no fungal elements. The analysis attributed the recurrence to contaminated skincare products. Given the prior diagnosis of tinea incognito with folliculitis, the new lesions were preliminarily diagnosed as atypical tinea with folliculitis, despite negative mycology. Antifungal therapy was initiated first to achieve partial improvement. The subsequent addition of antibacterial treatment led to complete resolution, confirming the accuracy of the recurrent diagnosis. Preventive measures: To prevent reinfection from fomites, the patient was instructed to discard all previously used skincare products and sterilize towels and wash basins by boiling.
3. Antifungal treatment strategies: Common pathogens that cause TI include *Trichophyton rubrum*, *T. mentagrophytes*, *Epidermophyton floccosum*, *Microsporum canis*, and *M. gypseum*.^[2]^ Itraconazole (oral), a broad-spectrum triazole antifungal agent, is effective against superficial mycoses.^[5]^ Topical naftifine–ketoconazole cream functions as a dual-action antifungal agent with efficacy against diverse pathogens.^[6]^ Combination therapy (systemic and topical) achieved optimal treatment outcomes in most cases.
4. Management of folliculitis^[7]^: The primary pathogen causing folliculitis is *Staphylococcus aureus*. Oral erythromycin, a macrolide antibiotic, targets Gram-positive bacteria and is indicated for treating acne/folliculitis. Topical fusidic acid cream demonstrates efficacy against Gram-positive cocci in conditions such as impetigo and furunculosis. The combined regimen (oral erythromycin with topical fusidic acid) provided comprehensive Gram-positive coverage.
5. Empirical therapy in clinical practice: Meticulous lesion assessment and timely empirical antifungal and/or antibacterial therapy are essential for TI and folliculitis, particularly when immediate therapeutic decisions are required while awaiting delayed microbiological results.

## Acknowledgments

We thank the patients for their participation in the study.

## Author contributions

**Writing – original draft:** Nenghan Zhang.

**Writing – review & editing:** Ruifeng Zhang.
